# A novel integrated MADM method for design concept evaluation

**DOI:** 10.1038/s41598-022-20044-7

**Published:** 2022-09-23

**Authors:** Zhe Chen, Peisi Zhong, Mei Liu, Qing Ma, Guangyao Si

**Affiliations:** 1 Advanced Manufacturing Technology Centre, Shandong University of Science and Technology, Qingdao, China; 2 Shandong Jiaotong University, Jinan, China; 3 College of Mechanical and Electronic Engineering, Shandong University of Science and Technology, Qingdao, China; 4 Yantai Research Institute and Graduate School of Harbin Engineering University, Yantai, China; 5 School of Minerals and Energy Resources Engineering, University of New South Wales, Sydney, 2052 Australia

**Keywords:** Applied mathematics, Information technology

## Abstract

Design concept evaluation plays a significant role in new product development. Rough set based methods are regarded as effective evaluation techniques when facing a vague and uncertain environment and are widely used in product research and development. This paper proposed an improved rough-TOPSIS method, which aims to reduce the imprecision of design concept evaluation in two ways. First, the expert group for design concept evaluation is classified into three clusters: designers, manufacturers, and customers. The cluster weight is determined by roles in the assessment using a Multiplicative Analytic Hierarchy Process method. Second, the raw information collection method is improved with a 3-step process, and both design values and expert linguistic preferences are integrated into the rough decision matrix. The alternatives are then ranked with a rough-TOPSIS method with entropy criteria weight. A practical example is shown to demonstrate the method’s viability. The findings suggest that the proposed decision-making process is effective in product concept design evaluation.

## Introduction

As companies pay more attention to R&D in the current technology-driven era, new product development (NPD) has been recognized as a significant issue to deal with market competition. Design concept evaluation is a critical phase in NPD. Generally, various concepts are proposed and decision makers are assigned to select the best one for further development. Once the decision is made, the R&D of the product and over 70% of the cost are determined. Compensating for problems caused by a poor design concept at later stages is very difficult^1^. Because of the interconnected factors, the process for complex products is even harder^2^, and the loss caused by an incorrect decision will be considerable. Thus, the stage of design concept evaluation is both essential and challenging^3^. To reduce the subjective bias caused by individual preference, group decision-making is implemented. Meanwhile, as the evaluation attributes are multiple and complex, multiple attribute decision-making (MADM) methods are receiving considerable interest in design concept evaluation^4^.

Most of the studies in design concept evaluation concentrate on improving the criteria weight determination and the assessment method. In these studies, customers participate in the assessments as experts, and they give their preferences to each design scheme according to various attributes. As the customers’ preferences are usually vague and uncertain, researchers have used various means to overcome the imprecision. For instance, fuzzy method^5,6^ and grey theory^7^ are applied widely in design concept evaluation. Geng^1^ introduced the concept of a vague number to describe linguistic variables, and other research used an interval 2-tuple linguistic to describe the uncertainty and imprecision of the decision makers’ preferences^8^. Compared to the vague theory, the rough set theory is more feasible in design concept evaluation.

Rough set was introduced by Pawlak, and widely used in the MADM method after it was first proposed^9^. Zhai^10^ used rough numbers (RNs) to quantify the vagueness of raw information, and proposed an integrated method based on rough set and grey relation analysis. Zhu^11^, Chen^4^, Tiwari^12^ and Song^13^ also converted the raw data to intervals using rough set theory. Shidpour^14^ constructed two decision matrices using a rough set and a fuzzy set. In his study, the triangular fuzzy numbers (TFNs) and the rough numbers are converted into crisp numbers by specific methods. After the criteria weight is determined by the extent analysis method^15^, the design concepts are computed by measuring the distance between the alternative interval vectors and the positive and negative ideal reference vectors. Recently, rough-TOPSIS^16^, rough-VIKOR^12^ and rough-AHP^13^ methods have also been implemented in design concept evaluation.

**Figure 1:**
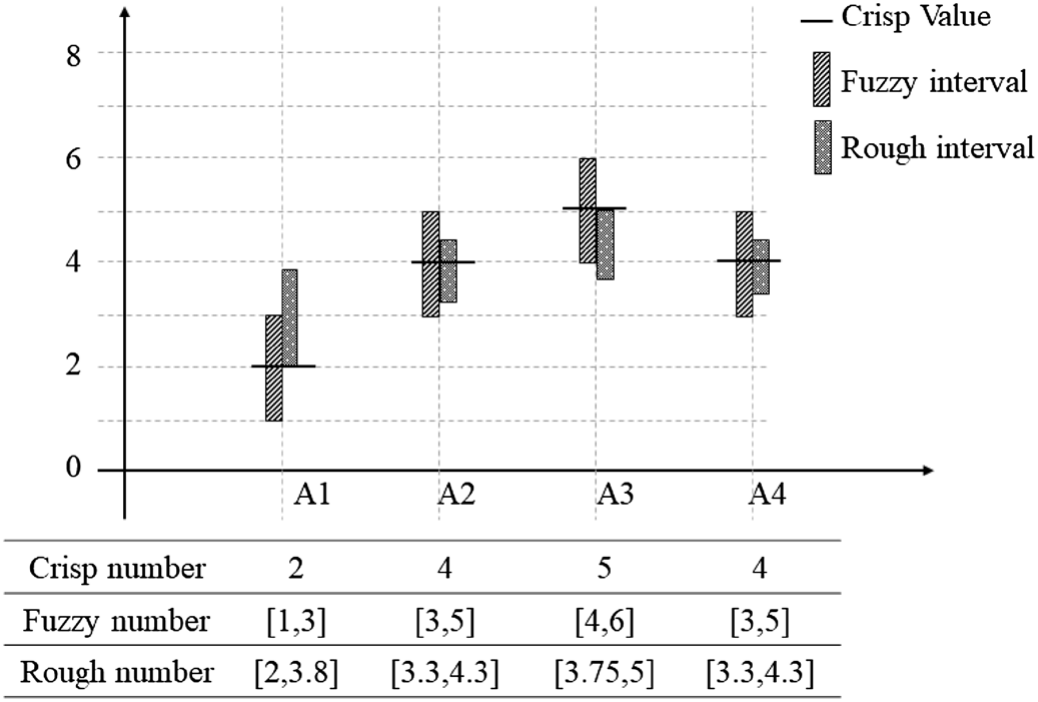
Comparison of crisp number, fuzzy number and rough number approaches.

Compared with the other extensions of the fuzzy set, the rough set does not need further individual judgment information in the decision matrix building^4^. In other words, the method is more objective compared to other fuzzy logic methods. The rough set also shows excellent performance in demonstrating the vagueness of human beings. According to cognition theory, the linguistic information of decision makers is considered as a preference close to the description (eg. close to very good, close to extremely poor). The distribution range of decision makers’ judgments can be illustrated as an interval on the axis. Furthermore, the information presents a normal distribution, and the center does not exactly correspond to the crisp integer. As is shown in Fig. 1, the fuzzy numbers are simply expanded the same distance towards each side. Unlike fuzzy numbers, the rough set method establishes the interval via a series of rigorous equations. The information treatment of the rough set is very similar to human cognition behaviors. Because of the outstanding performance in design concept evaluation, the rough-TOPSIS method is applied in our study. Focusing on the characteristic of design concept evaluation, two modifications are developed in our research: the expert weight determination and the integration of information from different sources.

In previous studies, the method for determining the criteria weight usually plays a significant role in MADM problems. In contrast, expert weight is seldom mentioned in design concept evaluation. When the optimal product concept design must be selected from several design schemes, the decision-making organizers usually assign a group of customers to give their preferences, and the experts are viewed as a group with homogeneous weights during the assessment^17^. However, it does not mean that the expert weight is not important in the assessment. The expert weight determination is also an important component in the MADM structure^18^. Nevertheless, the design concept evaluation criteria usually include customer needs, R&D-specific parameters, and business objectives^19^. The expert group should include not only the customers but also professional R&D experts.

The decision matrix is integrated from the information collected according to the criteria. For some attributes, the information can be collected in two ways: the parameter value from the R&D department and the perception information from the expert preference. Taking the attribute “size” as an example, it can be judged from two perspectives: the design parameter values (such as 1.21 m) show the practical length, width, and height of the product; and the user subjective evaluation (such as “very spacious”) shows the expert individual reaction to the attribute size. Obviously, both design values and customer preference values are critical to the design concept evaluation and should be considered in the assessment.

This work attempts to mitigate the uncertainty and imprecision in the rough-TOPSIS method for product design concept evaluation. The improvements are conducted in two ways.

1. The expert group is formed by three clusters: experienced designers, manufacturers and customers. The cluster weight of each cluster is determined by the Multiplicative AHP method.
2. The raw information collection method is improved with a 3-step process, and the decision matrix is integrated by the information from both design values and customer preference values.

The remaining sections of the paper are arranged as follows. Section “State of the art” briefly reviews the related basic notions. Section “Methodology” details the proposed method. A real-life example is then given in Sect. “Case study”. Section “Conclusion” contains the concluding remarks.

## State of the art

This section includes four parts. The MADM studies in design concept evaluation are generally reviewed in Sect. “MADM in design concept evaluation”, then the rough set and rough-TOPSIS method are described as the methods we apply in Sect. “Rough set and the rough-TOPSIS method”. Sections “Expert weight determination” and “The information from design values and expert preference” describe the modification we made based on the rough-TOPSIS method.

### MADM in design concept evaluation

In NPD, enterprises are keen to win market share by improving their design concept. A common way to improve the design concept is to select the most appropriate concept from various suggested concepts. Hence, design concept evaluation is proposed as a significant phase to make the decision for subsequent design activities. Some simple design concept evaluation methods are proposed as quick decision-making approaches, such as SWOT analysis^20^, house of quality^21^, Pugh chart^22^, and screening matrix^23^. Nevertheless, if the product is complex, there are numerous and interconnected decision factors and it is difficult to make a correct decision relying on simple methods. Experts then implement typical MADM approaches to solve design concept selection problems. Ayağ^24^ introduced an analytic network process (ANP) based method in concept selection considering the needs of both customers and the company. From customer requirements and design characteristics, Lin^25^ proposed a hybrid method based on the Analytic Hierarchy Process (AHP) and the Technique for Order of Preference by Similarity to Ideal Solution (TOPSIS) method to help designers achieve an effective concept selection. Akay^7^ integrated grey theory and fuzzy set to solve both grey type and fuzzy type uncertainties in design concept evaluation. Takai^26^ decomposed the quality function deployment (QFD) matrices simultaneously and reconstructed a target costing and perception-based concept evaluation method for complex and large-scale systems. Moreover, other general evaluation models are also applied in design concept evaluation, such as VlseKriterijumska Optimizacija I KOmpromisno Resenje (VIKOR)^27^, elimination et Choice translating reality (ELECTRE)^28^, preference ranking organizational method for enrichment evaluation (PROMETHEE)^29^ and evaluation based on distance from average solution (EDAS)^30^.

Recently, studies have concentrated on the uncertainty of the decision environment. The crisp number has some limitations in expressing the vagueness of the raw data. Fuzzy set (FS) theory was proposed by Zadeh^31^ to deal with vagueness involved in decision-making problems, and various fuzzy data types are applied in uncertain environment identification. Zadeh^32^ introduced the Type-2 fuzzy sets and interval-valued fuzzy sets. Garibaldi^33^ revised the Type-2 fuzzy sets and proposed nonstationary fuzzy sets. Atanassov^34^ introduced the intuitionistic fuzzy set to describe the uncertainty of the linguistic information, and Xu^35^ proposed a related geometric aggregation operator. Rodríguez^36^ proposed the hesitant fuzzy linguistic term sets to increase the flexibility and richness of linguistic elicitation. Correspondingly, fuzzy set integrated MADM models are implemented in uncertain environments, such as fuzzy VIKOR^37^ and fuzzy TOPSIS^38^. Nevertheless, the boundary of fuzzy numbers needs to be determined subjectively before the assessment process, and this may affect the result of the evaluation^17,39^.

### Rough set and the rough-TOPSIS method

Rough set theory is another vital mathematical data analysis approach in an uncertain environment, normally expressed as an interval named the rough number (RN). It is another extension of fuzzy sets. In contrast to the fuzzy number, rough number treats the uncertain information without an external pre-setting interval boundary or additional membership function and distribution forms^4^. Zhai^10,40^ first applied the method in design concept evaluation, where the rough set is widely used in product concept selection. Rough set theory is suitable for raw data treatment, commonly integrated with the general MADM method. We discussed the superiority of the rough set in the introduction section, and this is also true in practice. Of the top 10 papers cited from 2000 to 2022 selected from the Web of science database using the keywords “design concept evaluation”, half of them (5 papers) used the RN integrated method. Zhu^27^ proposed an RN based AHP criteria model and an RN-TOPSIS evaluation method in lithography tool selection. Song^13^, Shidpour^14^ and Zhu^16^ integrated RN with AHP or fuzzy-AHP, and Tiwari^12^ integrated RN with VIKOR. Here we briefly review the basic theory of RNs.

A rough set contains a lower approximation and an upper approximation, defined as two target sets. In the RN, the lower approximation and the upper approximation are represented as the conservative and liberal target set, respectively. Thus, an RN can be set as an interval. The rules of RNs are presented as follows:

The symbol $U$ represents the universe including all the objects in the information table. Assume the object is a set $R$ constructed by $n$ classes. The set can be described as $R=\left\{{C}_{1},{C}_{2},{C}_{3},\dots,{C}_{n}\right\}$, where ${C}_{1}<{C}_{2}<{C}_{3}<\cdots <{C}_{n}$. For ${C}_{i}\in R, 1\le i\le n$, ${C}_{i}$ can be expressed as an interval ${C}_{i}=[{C}_{li},{C}_{ui}]$, provided that ${C}_{li}<{C}_{ui}$ and $1\le i\le n$. Here ${C}_{li},{C}_{ui}$ represent the lower and the upper limit, respectively. Thus $\forall Y\in U$:

The lower approximation can be defined as the equation below.

1 $$\begin{array}{*{20}c} {\underline{Apr} \left( {C_{i} } \right) = \cup \left\{ {Y \in U/R\left( Y \right) \le C_{i} } \right\}} \\ \end{array}$$

While the upper approximation can be defined as the following equation.

2 $$\begin{array}{*{20}c} {\overline{Apr} \left( {C_{i} } \right) = \cup \left\{ {Y \in U/R\left( Y \right) \ge C_{i} } \right\}} \\ \end{array}$$

The boundary region of ${C}_{i}$ is determined as:

3 $$\begin{array}{c}Bnd\left({C}_{i}\right)=\cup \left\{Y\in U/R\left(Y\right)\ne {C}_{i}\right\}=\left\{Y\in U/R\left(Y\right)>{C}_{i}\right\}\cup \left\{Y\in U/R\left(Y\right)<{C}_{i}\right\}\end{array}$$

Thus, the vague class ${C}_{i}$ in the universe $U$ can be represented by the RN. If we use $\underline{Lim}({C}_{i})$ and $\overline{Lim}\left({C}_{i}\right)$ to express the upper and the lower limit of the RN, the $RN\left({C}_{i}\right)$ can be defined as:

4 $$\begin{array}{c}RN\left({C}_{i}\right)=\left[\underline{Lim}\left({C}_{i}\right),\overline{Lim}\left({C}_{i}\right)\right]=\left[\frac{1}{{M}_{L}}\sum R\left(Y\right)\left|Y\in \underline{Apr}\left({C}_{i}\right), \frac{1}{{M}_{U}}\sum R\left(Y\right)\right|Y\in \overline{Apr}\left({C}_{i}\right)\right]\end{array}$$

where ${M}_{L}$/${M}_{U}$ is the number of elements in $\underline{Apr}\left({C}_{i}\right)$ / $\overline{Apr}\left({C}_{i}\right)$, and the interval of the $RN\left({C}_{i}\right)$ is computed as:

5 $$\begin{array}{c}RBnd\left({C}_{i}\right)=\overline{Lim}\left({C}_{i}\right)-\underline{Lim}\left({C}_{i}\right)\end{array}$$

In a rough set, the $RBnd\left({C}_{i}\right)$ shows the vagueness of the class. Although the rough set proposed a model dealing with the uncertain environment, the model is not a complete decision-making framework^12^. That is why the most frequently cited papers in design concept evaluation are RN integrated methods but not the rough set model. In our study, we integrated the rough set and TOPSIS method. The TOPSIS method is one of the most well-known and widely used methods in MADM problems^41^. The method is based on the idea of compromise where the alternatives are ranked by calculating the closeness index between the alternative and the ideal solution.

**Figure 2:**
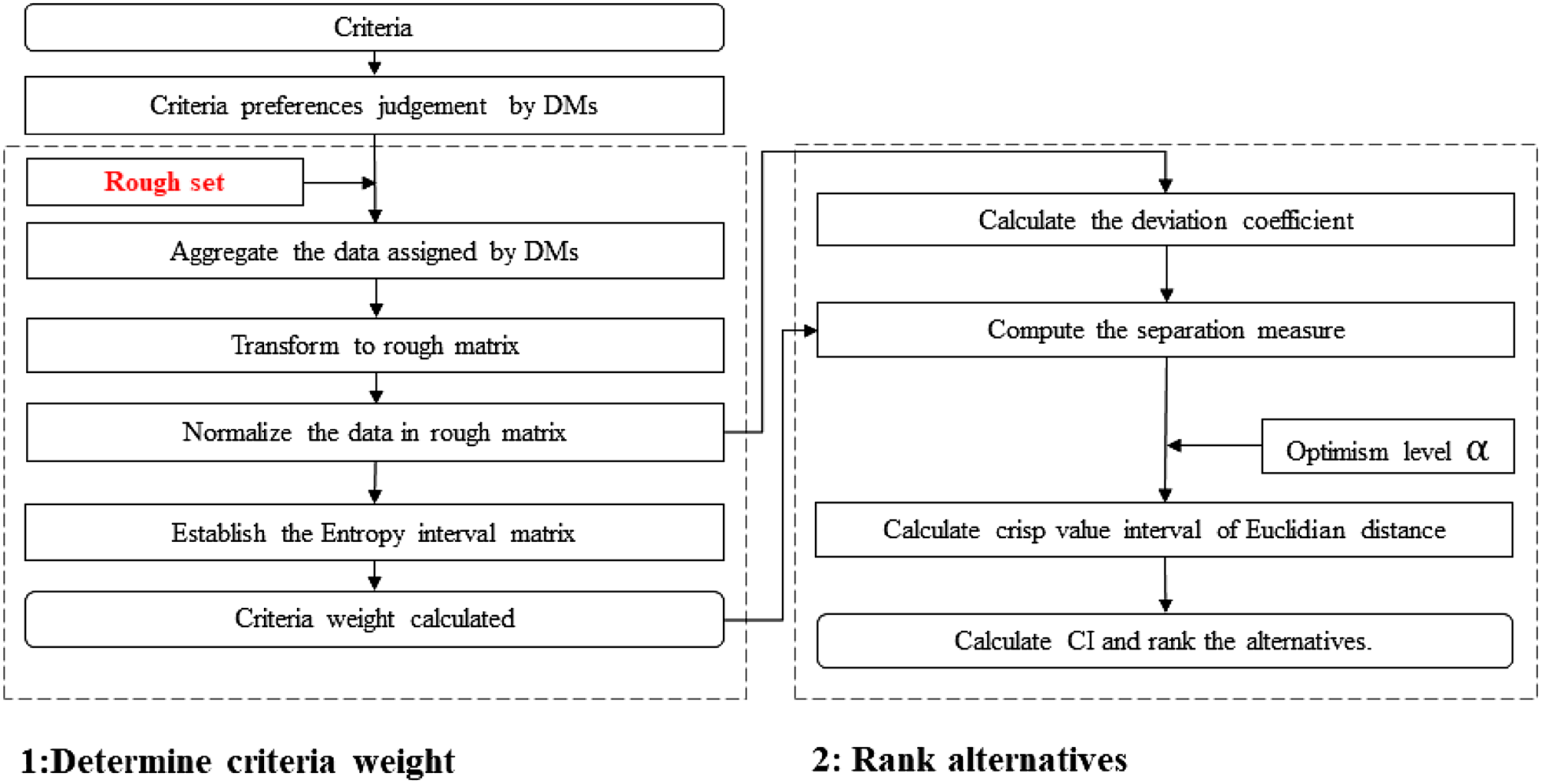
The process of the rough-TOPSIS method.

The rough-TOPSIS method is an efficient method in design concept evaluation. Song^13^ implemented the rough-TOPSIS method in design concept evaluation, and the criteria weight was determined by a rough AHP method. Chen^4^ improved the criteria weight determination method and integrated the rough entropy criteria weight, rough-TOPSIS method and the preference selection index (PSI) method in his study. We implement the rough entropy criteria and rough-TOPSIS method in our study. The process of the rough-TOPSIS method is shown in Fig. 2. There are two main steps in the design concept evaluation.

#### STEP 1. Determine criteria weight

Assume $p$ experts are assigned to evaluate $m$ alternatives according to $n$ criteria using linguistic information. The information is converted into crisp numbers by the scale index^4,13^. The criteria weight determination method is proposed by Lotfi^42^.

After that, the crisp numbers are converted into rough numbers by Eqs. (1)–(5), and a rough decision matrix $A$ is then constructed.

6 $$\begin{array}{c}A=\left[\begin{array}{cc}\begin{array}{cc}\left[{a}_{11}^{l},{a}_{11}^{u}\right]& \left[{a}_{12}^{l},{a}_{12}^{u}\right]\\ \left[{a}_{21}^{l},{a}_{21}^{u}\right]& \left[{a}_{22}^{l},{a}_{22}^{u}\right]\end{array}& \begin{array}{cc}\cdots & \left[{a}_{1n}^{l},{a}_{1n}^{u}\right]\\ \cdots & \left[{a}_{2n}^{l},{a}_{2n}^{u}\right]\end{array}\\ \begin{array}{cc}\vdots & \vdots \\ \left[{a}_{m1}^{l},{a}_{m1}^{u}\right]& \left[{a}_{m2}^{l},{a}_{m2}^{u}\right]\end{array}& \begin{array}{cc}& \vdots \\ \cdots & \left[{a}_{mn}^{l},{a}_{mn}^{u}\right]\end{array}\end{array}\right] \end{array}$$

Here we use interval $[{a}_{ij}^{l},{a}_{ij}^{u}]$ to illustrate the rough number of the $i$ ^th^ alternative and the $j$ ^th^ attribute.

Then the interval is normalized by a linear scale transformation below:

7 $$\begin{array}{c}{p}_{ij}^{l}=\frac{{a}_{ij}^{l}}{{\sum }_{i=1}^{m}{a}_{ij}^{u}} \end{array}$$

8 $$\begin{array}{c}{p}_{ij}^{u}=\frac{{a}_{ij}^{u}}{{\sum }_{i=1}^{m}{a}_{ij}^{u}} \end{array}$$

where [${p}_{j}^{l},{p}_{j}^{u}$] denotes the interval relative criterion importance rating. $\left[{Q}_{i}^{l}, {Q}_{i}^{u}\right]$ satisfies the properties for ${p}_{i}$, within estimation joint probability distribution $P$. The lower limit ${Q}_{i}^{l}$ and the upper limit ${Q}_{i}^{u}$ can be computed by Eqs. (9) and (10).

9 $${Q_{i}^{l} = \min \left\{ {\frac{{ - \left[ {\mathop \sum \nolimits_{{j = 1}}^{m} p_{{ij}}^{l} \ln \left( {p_{{ij}}^{l} } \right)} \right]}}{{\ln \left( m \right)}},\frac{{ - \left[ {\mathop \sum \nolimits_{{j = 1}}^{m} p_{{ij}}^{u} \ln \left( {p_{{ij}}^{u} } \right)} \right]}}{{\ln \left( m \right)}}} \right\}}$$

10 $${Q_{i}^{u} = \max \left\{ {\frac{{ - \left[ {\mathop \sum \nolimits_{{j = 1}}^{m} p_{{ij}}^{l} \ln \left( {p_{{ij}}^{l} } \right)} \right]}}{{\ln \left( m \right)}},\frac{{ - \left[ {\mathop \sum \nolimits_{{j = 1}}^{m} p_{{ij}}^{u} \ln \left( {p_{{ij}}^{u} } \right)} \right]}}{{\ln \left( m \right)}}} \right\}}$$

Special conditions: If ${p}_{ij}^{l}=0$, ${p}_{ij}^{l}ln\left({p}_{ij}^{l}\right)$ is defined as 0; correspondingly if ${p}_{ij}^{u}=0$, ${p}_{ij}^{l}ln\left({p}_{ij}^{l}\right)$ is defined as 0. The criteria weight of the decision matrix can be illustrated as an interval, and the lower and the upper limit is determined by the equations below:

11 $$\begin{array}{c}{W}_{j}^{l}=\frac{1-{Q}_{j}^{u}}{{\Sigma }_{i=1}^{n}\left(1-{Q}_{i}^{u}\right)} \end{array}$$

12 $$\begin{array}{c}{W}_{j}^{u}=\frac{1-{Q}_{j}^{l}}{{\Sigma }_{i=1}^{n}\left(1-{Q}_{i}^{l}\right)} \end{array}$$

where ${W}_{j}^{l}$ and ${W}_{j}^{u}$ denote the lower and the upper limit respectively, the weight of attribute $j$ can be written as ${[W}_{j}^{l}, {W}_{j}^{u}]$, $1\le j\le n$.

#### STEP 2. Rank alternatives

In this step, the rough decision matrix should be normalized before the ranking process. Vector normalization (VN), sum normalization (SN) and min–max normalization (MMN) are popularly used normalization methods. Chen^43^ compared the three normalization methods: among the three normalization methods, the VN and SN will not change the diversity of attribute data, and VN is suggested in the TOPSIS method. The decision matrix $A$ can be normalized by the equations below:

13 $$\begin{array}{c}{r}_{ij}^{-}=\frac{{a}_{ij}^{l}}{\sqrt{{\sum }_{i=1}^{m}{\left({a}_{ij}^{u}\right)}^{2}}}\end{array}$$

14 $$\begin{array}{c}{r}_{ij}^{+}=\frac{{a}_{ij}^{u}}{\sqrt{{\sum }_{i=1}^{m}{\left({a}_{ij}^{u}\right)}^{2}}}\end{array}$$

where $\left[{r}_{ij}^{-},{r}_{ij}^{+}\right]$ denotes the normalized rough number $[{a}_{ij}^{l},{a}_{ij}^{u}]$ in the decision matrix $A$. The normalized matrix $R$ can be written as

15 $$\begin{array}{c}R={\left(\left[{r}_{ij}^{-},{r}_{ij}^{+}\right]\right)}_{m\times n} \end{array}$$

For the benefit attribute, the upper bound is the positive ideal solution (PIS) and the lower bound represents the negative ideal solution (NIS); for the cost attributes, the upper bound represents PIS while the lower bound means NIS. The PIS and NIS of attribute $j$ can be shown as:

16 $$\begin{array}{c}PIS={r}^{+}\left(j\right)=\left\{\underset{i}{\mathrm{max}}\left({r}_{ij}^{+}\right),j\in \mathrm{Benefit\, attribute}; \underset{i}{\mathrm{min}}\left({r}_{ij}^{-}\right), j\in \mathrm{Cost\, attribute}\right\}\end{array}$$

17 $$\begin{array}{c}NIS= {r}^{-}\left(j\right)=\left\{\underset{i}{\mathit{min}}\left({r}_{ij}^{-}\right),j\in Benefit\, attribute; \underset{i}{\mathit{max}}\left({r}_{ij}^{+}\right),j\in Cost\, attribute\right\}\end{array}$$

The deviation coefficient representatives of PIS and NIS are shown in Table 1.

**Table 1:** Deviation coefficient of the attribute $j$.

Deviation coefficient	Lower bound	Upper bound
**Benefit attribute**		
Deviation to PIS	${d}_{ij}^{+l}={r}^{+}\left(j\right)-{r}_{ij}^{+}$	${d}_{ij}^{+u}={r}^{+}\left(j\right)-{r}_{ij}^{-}$
Deviation to NIS	${d}_{ij}^{-l}=-{r}^{-}\left(j\right)+{r}_{ij}^{+}$	${d}_{ij}^{-u}=-{r}^{-}\left(j\right)+{r}_{ij}^{-}$
**Cost attribute**		
Deviation to PIS	${d}_{ij}^{+l}=-{r}^{+}\left(j\right)+{r}_{ij}^{-}$	${d}_{ij}^{+u}=-{r}^{+}\left(j\right)+{r}_{ij}^{+}$
Deviation to NIS	${d}_{ij}^{-l}={r}^{-}\left(j\right)-{r}_{ij}^{+}$	${d}_{ij}^{-u}={r}^{-}\left(j\right)-{r}_{ij}^{-}$

Where the deviations to PIS and NIS are defined as $\left[{d}_{ij}^{+l},{d}_{ij}^{+u}\right]$ and $\left[{d}_{ij}^{-l},{d}_{ij}^{-u}\right]$.

After that, the deviation coefficient matrices need to be normalized again to compare with each other. For the PIS deviation coefficient interval $\left[{d}_{ij}^{+l},{d}_{ij}^{+u}\right]$,

18 $$\begin{array}{c}\left\{\begin{array}{c}{d}_{ij}^{{+}^{\mathrm{^{\prime}}}l}=\frac{{d}_{ij}^{+l}}{{\mathrm{max}}_{i=1}^{m}\left\{\mathrm{max}\left[{d}_{ij}^{+l},{d}_{ij}^{+u}\right]\right\}}\\ {d}_{ij}^{{+}^{\mathrm{^{\prime}}}u}=\frac{{d}_{ij}^{+u}}{{\mathrm{max}}_{i=1}^{m}\left\{\mathrm{max}\left[{d}_{ij}^{+l},{d}_{ij}^{+u}\right]\right\}}\end{array} \right. \end{array}$$

For the NIS deviation coefficient interval $\left[{d}_{ij}^{-l},{d}_{ij}^{-u}\right]$,

19 $$\begin{array}{c}\left\{\begin{array}{c}{d}_{ij}^{{-}^{\mathrm{^{\prime}}}l}=\frac{{d}_{ij}^{-l}}{{\mathrm{max}}_{i=1}^{m}\left\{\mathrm{max}\left[{d}_{ij}^{-l},{d}_{ij}^{-u}\right]\right\}}\\ {d}_{ij}^{{-}^{\mathrm{^{\prime}}}u}=\frac{{d}_{ij}^{-u}}{{\mathrm{max}}_{i=1}^{m}\left\{\mathrm{max}\left[{d}_{ij}^{-l},{d}_{ij}^{-u}\right]\right\}}\end{array}\right. \end{array}$$

where the normalized deviation coefficients to PIS and NIS are defined as $\left[{d}_{ij}^{{+}^{\mathrm{^{\prime}}}l},{d}_{ij}^{{+}^{\mathrm{^{\prime}}}u}\right]$ and $\left[{d}_{ij}^{{-}^{\mathrm{^{\prime}}}l},{d}_{ij}^{{-}^{\mathrm{^{\prime}}}u}\right]$.

The separation measure ${S}^{+}$ and ${S}^{-}$ are computed as the weighted deviation, denoting the dissimilarity of an information sequence of PIS and NIS values.

20 $$\begin{array}{c}\left\{\begin{array}{c}{S}_{i}^{+}=\left[{S}_{i}^{+l},{S}_{i}^{+u}\right]={\sum }_{j=1}^{n}\left[{W}_{j}^{l}, {W}_{j}^{u}\right]\times \left[{d}_{ij}^{{+}^{\mathrm{^{\prime}}}l},{d}_{ij}^{{+}^{\mathrm{^{\prime}}}u}\right]\\ {S}_{i}^{-}=\left[{S}_{i}^{-l},{S}_{i}^{-u}\right]={\sum }_{j=1}^{n}\left[{W}_{j}^{l}, {W}_{j}^{u}\right]\times \left[{d}_{ij}^{{-}^{\mathrm{^{\prime}}}l},{d}_{ij}^{{-}^{\mathrm{^{\prime}}}l}\right]\end{array}\right. \end{array}$$

The crisp value of rough interval $[{S}_{i}^{-}, {S}_{i}^{+}]$ is transformed to:

21 $$\begin{array}{c}\left\{\begin{array}{c}{S}_{i}^{+*}=\left(1-\mathrm{\alpha }\right){S}_{i}^{+l}+\mathrm{\alpha }{S}_{i}^{+u}\\ {S}_{i}^{-*}=\left(1-\mathrm{\alpha }\right){S}_{i}^{-l}+\mathrm{\alpha }{S}_{i}^{-u}\end{array}\right. \end{array}$$

where $\alpha$ represents an optimism level, valued in the interval $[\mathrm{0,1}]$, and for a rational condition, $\alpha = 0.5$, $\alpha >0.5$ and $\alpha <0.5$ denote the optimistic and the pessimistic selection of the assessment manager.

The closeness indices ($CIs$) are calculated to rank the alternatives:

22 $$\begin{array}{c}{CI}_{i}=\frac{{S}_{i}^{-*}}{{S}_{i}^{+*}+{S}_{i}^{-*}} \end{array}$$

The optimistic alternative is close to the PIS and far from the NIS, which means the value of ${S}_{i}^{+*}$ is as small as possible while ${S}_{i}^{-*}$ is as large as possible. From Eq. (22), we can select the best candidate is the alternative approach to 1. The alternatives can be ranked by the value of $CIs$.

### Expert weight determination

**Figure 3:**
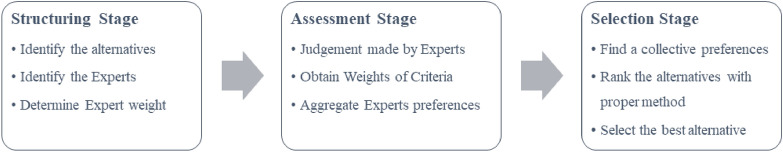
General MADM evaluation process.

As shown in Fig. 3, expert weight determination is a critical phase in the structuring stage. However, 59% of the top cited papers in group decision-making (GDM) omitted this phase^18^. We examined the top cited 8 papers in design concept evaluation^1,11–14,27,40^. None of them mentioned expert weight, and experts are treated as homogeneous individuals.

Although the rough-TOPSIS method has revealed its outstanding performance in design concept evaluation, biases may still appear in the assessment without the expert weight consideration. As experts have an important role, various studies about the expert weights are carried out in other fields of MADM problems^44^. The weights of experts depend on their background and experience^45^. Both subjective and objective methods have been applied in expert weight determination for years^44^. Subjective expert weights rely on the supervisors’ preferences or the pairwise comparison between experts. Multiplicative AHP^46^, simple multi-attribute rating technique (SMART)^47^ and Delphi^48^ are implemented to determine expert weights. All three methods are determined by comparing the attributes by experts in pairs. Objective methods for determining the expert weight depend on the proposed information. One way is to measure the expert preference for the aggregated decision^49,50^. The expert whose decision has minimum distance to the ideal solution gets the highest weight. Another way is to maximize the group consensus. Expert weights are given to make the judgments of experts closer^51,52^.

Before the assessment, the decision maker group needs to be fixed in advance. Although only customers are mentioned as experts in some studies^53–55^, it is not recommended to form the expert group with only customers. Generally, user-centered design can help companies satisfy consumers’ preferences^56^, and the customer plays a role as a decision maker in product development evaluation. The project manager expects to organize a decision-making group with experienced customers. However, using many customers may not work well in the assessments^45^. Some studies suggest that the experts in the R&D department should be selected as the experts because they are much more familiar with the criteria^16^, including product attractiveness, manufacturing, maintenance, cost, and time to market^19^. Thus, it is common to form the decision-making group with both consumers and expert producers^57^. In a previous study, the experts were recruited from amongst designers, manufacturers and customers, and a cluster-based expert weight determination method was proposed in the 2-tuple linguistic environment. The case study part of that paper found the variances of experts in each group were very small (Designer cluster: 0.32%, Manufacturer cluster: 0.35%, Consumer cluster: 1.15%)^45^, and widely considered statistically insignificant. It mainly occurs because the experts in the same cluster have a similar background and interests, and their preferences are thus probably similar. Hence, experts are divided into the customer cluster, the designer cluster and the manufacturer cluster, and to simplify the expert weight determination process, the decision makers in each cluster are viewed as homogeneous experts. In our study, the cluster weights are determined by the Multiplicative AHP method.

### The information from design values and expert preference

Design values and expert preference are the two key pieces of information in design concept evaluation, especially for customer-involved products^58^. In terms of an attribute, the designers provide the value of the parameter of the attribute considering function, usability, cost, construction, etc., while the experts give their individual views on the attribute. Both types of information are critical and none of them can be discarded to develop a good product.

Studies combining the information from the designer and customers have been carried out recently. Yang^59^ proposed an assessment method that integrated fuzzy decision and fuzzy cognitive map, and evaluated the experiences of both designers and customers. Qi^17,58,60^ made great efforts in design concept evaluation and integrated both sources of information in decision-making, proposed an evaluation model by integrating important levels and design features, and named the rough distance to redefined ideal solution method (RD-RIS) or the integrated ideal solution definition approach (I-ISD)^17^. The model integrated both design values and customer preference values, and ranked the alternatives based on the compromise theory. In the Qi study, design values and expert preferences were obtained separately. Design values were provided by the designers while the expert preferences were constructed from the customers’ preferences. In the designers’ view, the best concept can satisfy the design constraints in a functional way. In expert preferences, the importance of each criteria is categorized into three levels: most important attributes, medium important attributes and less important attributes. Hence, a 6-option rule (benefit & most important, benefit & medium important, benefit & less important, cost & most important, cost & medium important, cost & less important) ideal solution method is defined integrating design values and preference values. Preference values are only relevant in option selection, where the ranking of alternatives depends on the corresponding design values. Compared with design values, expert preferences illustrate the individual subjective feeling on the corresponding attribute. On one hand, the expert preferences are obtained from their individual judgments, and they are not as precise as design values. On the other hand, expert preferences may reflect the acceptance of the design scheme, and reveal important implications for product R&D. Moreover, in real-life cases, only some of the attributes can be evaluated by the corresponding design values, as attributes such as “user acceptance” are not available to measure with design values. Last, the preference important level may be confused. In the case study section of this study, the criteria weight of expert preference information is ${W}_{j}=\left\{{W}_{1}=0.3772, {W}_{2}=0.1608,{W}_{3}= 0.2250, {W}_{4}=0.2298\right\}$, and the gaps among the criteria weights are obvious, as shown in Eq. (23).

23 $$\begin{array}{c}{W}_{1}\left(Most \, important\right)\gg {W}_{4}{\approx W}_{3}\left(\mathrm{Medium \, important}\right)\gg {W}_{2}\left(Less \, important\right) \end{array}$$

However, if there are more attributes in the criteria index, and the deviations among the attribute weights are ambiguous, it is difficult to define the importance level of each attribute.

In our study, to maintain the information both from design values and expert preferences, data from both sources are integrated to form a new decision matrix.

## Methodology

**Figure 4:**
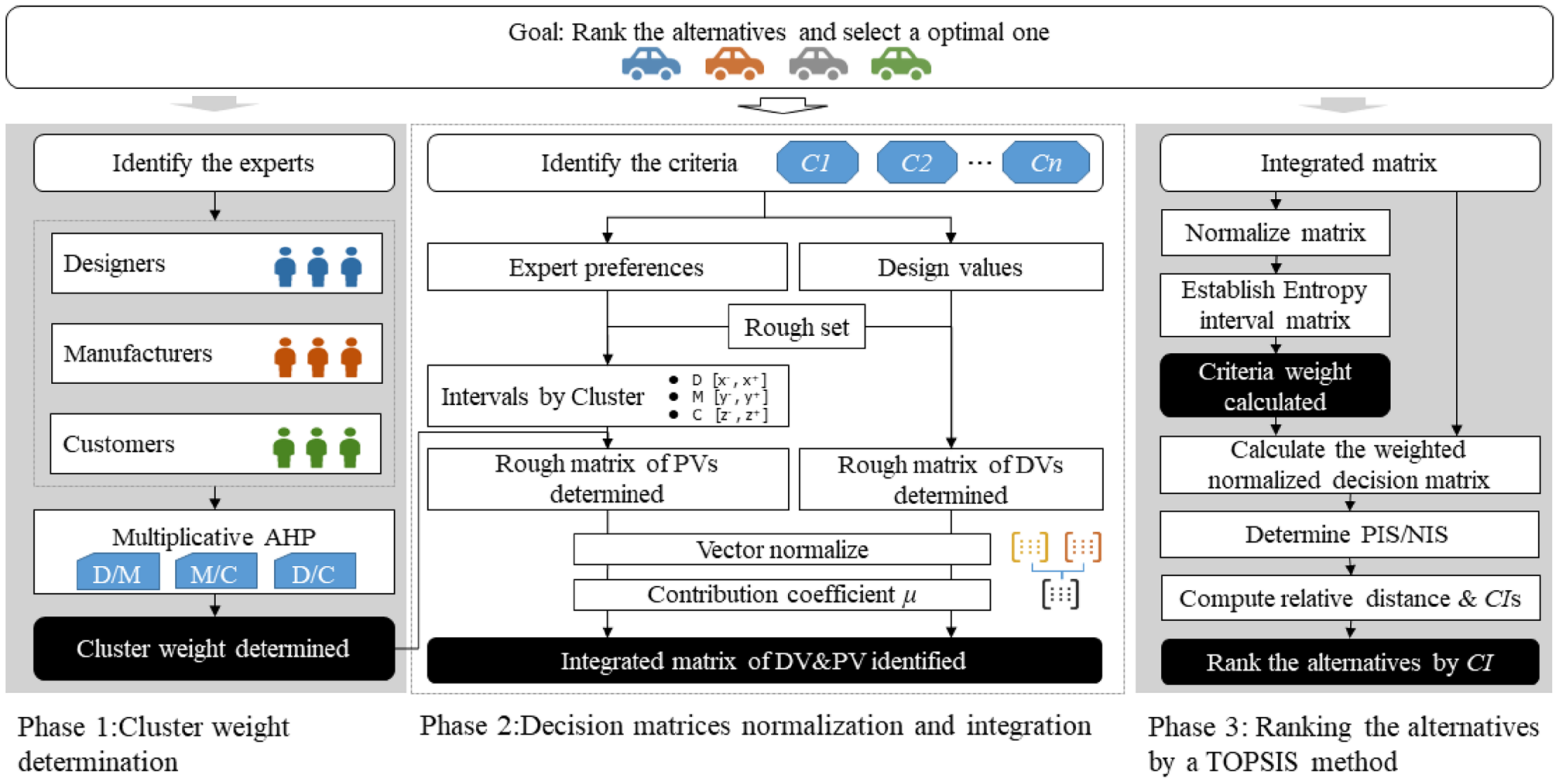
Framework of the proposed rough-TOPSIS method.

The purpose of design concept evaluation is to select an optimal design scheme from the proposed alternatives. To make the decision precise, a novel evaluation framework is proposed. The framework is constructed with three components as shown in Fig. 4. The alternatives, experts and the criteria are presented in Sect. 3.2. In phase 1, the experts are divided into three groups: the designer cluster, the manufacturer cluster and the customer cluster. The cluster weights are also determined by a Multiplicative AHP method in this phase. A 3-step information integration process is then conducted in phase 2. Both decision matrices of design values and preference values are converted into intervals according to the rough set theory, and after being normalized by the vector normalization method, the design values and preference values are integrated. By then, the pre-treated information matrix is identified. In the third phase, criteria weight is determined by a rough entropy method. The alternatives are then ranked by a rough-TOPSIS evaluation method. The details of the proposed method are presented in Sect. 3.2 to Sect. 3.4.

Assume there are $n$ criteria $C=\left\{{C}_{1},{C}_{2},\dots,{C}_{n}\right\}$ and $m$ design concept alternatives $A=\{{A}_{1},{A}_{2},\dots,{A}_{m}\}$ in an assessment. The design values of the assessment are obtained from the R&D department. The expert preference values are gathered from their preferences. Assume $s$ expert decision makers are assigned to give their linguistic preference {*extremely poor, very poor, poor, neutral, good, very good, extremely good*}, and the preferences are then converted into crisp numbers $\{1, 2, 3, 4, 5, 6, 7\}$, respectively.

### Phase 1: Determine cluster weights

As introduced in Sect. “Expert weight determination”, three expert groups are established: the designer cluster (DC), the manufacturer cluster (MC) and the customer cluster (CC). We implemented the Multiplicative AHP method to determine the expert cluster weight, and we set the three clusters as the alternatives in this section. The method follows the approach of Honert^61^. The details of the expert cluster weight determination method in our study are as follows.

To evaluate the importance of each cluster, all the experts were asked to give their preference of the three clusters using specified words as shown in Table 2, where ${S}_{\alpha }$ and ${S}_{\beta }$ denote the preference of cluster $\alpha$ and $\beta$, respectively.

**Table 2:** Integer-valued cluster important judgment designating the gradations.

${\mathrm{S}}_{\mathrm{\alpha }}$ versus ${\mathrm{S}}_{\upbeta }$	Comparative judgment				
	Very strong importance	Strong importance	Definite importance	Weak importance	Indifference
Gradation index Value	− 8	− 6	− 4	− 2	0
${\mathrm{S}}_{\upbeta }$ versus ${\mathrm{S}}_{\mathrm{\alpha }}$	Very strong importance	Strong importance	Definite importance	Weak importance	Indifference
Gradation index Value	8	6	4	2	0

Assume there are z experts in the cluster $y$, ${G}_{y}$ is defined as cluster $y$, ${G}_{y}^{t}$ is the $t$th expert in cluster $y$, ${G}_{y}\in \left\{DC,MC,CC\right\}$, $1\le t\le z$. Then we use ${G}_{y}^{t}({G}_{\alpha }/{G}_{\beta })$ to denote the comparison ${S}_{\alpha }$ versus ${S}_{\beta }$ made by ${G}_{y}^{t}$. The average preference of the experts in ${G}_{y}$ is ${\delta }_{\alpha \beta y}$, and can be computed by the arithmetic mean:

24 $$\begin{array}{c}{\delta }_{\alpha \beta y}= \frac{1}{z}\sum\nolimits_{t=1}^{z}{G}_{y}^{t}({G}_{\alpha }/{G}_{\beta })\end{array}$$

${r}_{\alpha \beta y}$ is defined to estimate the preference ratio ${S}_{\alpha }$ versus ${S}_{\beta }$ determined by ${G}_{y}$. The calculating equation is:

25 $$\begin{array}{c}{r}_{\alpha \beta y}=\mathrm{exp}\left(\upgamma {\delta }_{\alpha \beta y}\right)\end{array}$$

where $\upgamma$ is a scale parameter, normally equal to $\mathrm{ln}2$. According to Lootsma^62^, we determine the approximate vector $p$ by the logarithmic least-squares method. The vector $p$ minimizes

26 $$\begin{array}{c}\sum\nolimits_{\alpha <\beta }\sum_{{G}_{y}\in \left\{DC,MC,CC\right\}}{\left(\mathrm{ln}{r}_{\alpha \beta y}-\mathrm{ln}{p}_{\alpha }+\mathrm{ln}{p}_{\beta }\right)}^{2}\end{array}$$

We define ${w}_{\alpha }=\mathrm{ln}{p}_{\alpha }$, ${w}_{\beta }=\mathrm{ln}{p}_{\beta }$ and ${q}_{\alpha \beta y}=\mathrm{ln}{r}_{\alpha \beta y}$. The function is converted into

27 $$\begin{array}{c}\sum_{\alpha <\beta }{{\sum \nolimits }_{{G}_{y}\in \left\{DC,MC,CC\right\}}\left({q}_{\alpha \beta y}-{w}_{\alpha }+{w}_{\beta }\right)}^{2}\end{array}$$

i.e.

28 $$\begin{array}{c}{w}_{\alpha }{\sum }_{\alpha =1,\alpha \ne \beta }^{3}{N}_{\alpha \beta }-{\mathrm{w}}_{\beta }{\sum }_{\beta =1,\beta \ne \alpha }^{3}{N}_{\alpha \beta }={\sum }_{\beta =1,\beta \ne \alpha }^{3}{\sum }_{{G}_{y}\in \left\{DC,MC,CC\right\}}{q}_{\alpha \beta y}\end{array}$$

where $\alpha =\mathrm{1,2},3$,${N}_{\alpha \beta }=1$. To reduce the bias made by experts, comparisons including self-judgments are no longer valid, which means there are only two comparisons among three clusters. After the algebraic manipulation, the equation can be reduced to:

29 $$\begin{array}{c}2{w}_{\alpha }-{\sum }_{\beta =1,\beta \ne \alpha }^{3}{w}_{\beta }={\sum }_{\beta =1,\beta \ne \alpha }^{3}{\sum }_{{G}_{y}\in \left\{DC,MC,CC\right\}}{q}_{\alpha \beta y}\end{array}$$

From Table 2, we can infer, for each variable, ${q}_{\alpha \beta y}=-{q}_{\beta \alpha y}$, ${G}_{\alpha \alpha }$ is empty, ${q}_{\alpha \alpha y}=0$ and ${\sum }_{\beta =1,\beta \ne \alpha }^{3}{\mathrm{w}}_{\beta }=0$. ${\mathrm{w}}_{\beta }$ can be computed as

30 $$\begin{array}{c}{w}_{\alpha }=\frac{1}{2}{\sum }_{\beta =1,\beta \ne \alpha }^{3}{\sum }_{y=1,y\ne \alpha }^{3}{q}_{\alpha \beta y}\end{array}$$

The expert weight ${p}_{\alpha }$ is

31 $$\begin{array}{c}{p}_{\alpha }=\mathit{exp}\left({w}_{\alpha }\right)={\prod }_{\beta =1,\beta \ne \alpha }^{3}{\prod }_{y=1,y\ne \alpha }^{3}\mathrm{exp}{\left(\frac{1}{2}\gamma \right)}^{{\delta }_{\alpha \beta y}}\end{array}$$

The cluster weight ${W}_{\alpha }$ can be determined as

32 $$\begin{array}{c}{W}_{\alpha }={p}_{\alpha }/\sum {p}_{\alpha }\end{array}$$

The weights of other clusters can be calculated accordingly.

### Phase 2: Normalize and integrate decision matrices

To improve the precision of the decision, we integrated both design values and preference values. Here is the 3-step process of the proposed method.

#### Step 1: Establish raw matrices

While we want to get the design value and preference value of each criterion, not every criterion can be evaluated in the form of a design value. If the criterion cannot be evaluated, we mark a “N/A” in the corresponding space, with an example shown in Table 3.

**Table 3:** Attributes defined in the real-life case.

Attribute	Specification	Attribute type	Design value	Expert preference
${C}_{1}$	⋯	Benefit	Crisp number	linguistic
${C}_{2}$	⋯	Benefit	N/A	linguistic
⋯				
${C}_{n}$	⋯	Cost	Crisp number	linguistic

Convert the design values into an interval matrix based on RNs. Equations (1)–(5) give the calculating method, and the matrix of design values is shown as follows:

33 $$\begin{array}{c}A=\left[\begin{array}{cc}\begin{array}{cc}\left[{a}_{11}^{l},{a}_{11}^{u}\right]& \left[{a}_{12}^{l},{a}_{12}^{u}\right]\\ \left[{a}_{21}^{l},{a}_{21}^{u}\right]& \left[{a}_{22}^{l},{a}_{22}^{u}\right]\end{array}& \begin{array}{cc}\cdots & \left[{a}_{1n}^{l},{a}_{1n}^{u}\right]\\ \cdots & \left[{a}_{2n}^{l},{a}_{2n}^{u}\right]\end{array}\\ \begin{array}{cc}\vdots & \vdots \\ \left[{a}_{m1}^{l},{a}_{m1}^{u}\right]& \left[{a}_{m2}^{l},{a}_{m2}^{u}\right]\end{array}& \begin{array}{cc}& \vdots \\ \cdots & \left[{a}_{mn}^{l},{a}_{mn}^{u}\right]\end{array}\end{array}\right]\end{array}$$

where $A$ represents the matrix of design values.$[{a}_{ij}^{l},{a}_{ij}^{u}]$ is the interval in the $i$th data sequence corresponding to the $j$th criterion of matrix $A$. If the design value of the attribute is not available (marked N/A), we mark a symbol “−” for substitution.

For the preference values, the experts in the same cluster commonly have a similar background, thus the experts in the same cluster are regarded as homogeneous individuals of equal importance. The cluster weights were computed in Sect. 3.1. Thus, we use the weighted average operator to determine the final interval after integrating all the experts.

Let ${\upnu }_{t}$ be the crisp value converted from the preference expert $t$. The expert preferences ${U}_{t}$ in ${G}_{y}$ can be converted into RNs according to Eqs. (1)–(5), expressed as:

34 $$\begin{array}{c}{U}_{t}=\left[{U}_{t}^{l},{U}_{t}^{u}\right]\end{array}$$

where ${U}_{t}^{l}=\overline{{\upnu }_{x}}, \forall {\upnu }_{x}\le {\upnu }_{t} 1\le x \le z$; ${U}_{t}^{u}=\overline{{\upnu }_{x}}, \forall {\upnu }_{x}\ge {\upnu }_{t} 1\le x \le z$. The interval of the cluster is:

35 $$\begin{array}{c}{U}_{y}=\left[{U}_{y}^{l},{U}_{y}^{u} \right]=\left[\frac{1}{z}\sum_{t=1}^{z}{U}_{t}^{l}, \frac{1}{z}\sum_{t=1}^{z}{U}_{t}^{u}\right]\end{array}$$

Considering the cluster weight ${W}_{y}$, the element in the preference value matrix can be determined as:

36 $$\begin{array}{c}{b}_{ij}=\left[{b}_{ij}^{l}, {b}_{ij}^{u} \right]=\left[{\sum }_{y=1}^{3}({U}_{y}^{l}\times {W}_{y}),{\sum }_{y=1}^{3}({U}_{y}^{u}\times {W}_{y})\right]\end{array}$$

The matrix $B$ of preference values is shown as follows:

37 $$\begin{array}{c}B=\left[\begin{array}{cc}\begin{array}{cc}\left[\begin{array}{cc}{b}_{11}^{l}& {b}_{11}^{u}\end{array}\right]& \left[\begin{array}{cc}{b}_{12}^{l}& {a}_{12}^{u}\end{array}\right]\\ \left[\begin{array}{cc}{b}_{21}^{l}& {b}_{21}^{u}\end{array}\right]& \left[\begin{array}{cc}{b}_{22}^{l}& {b}_{22}^{u}\end{array}\right]\end{array}& \begin{array}{cc}\cdots & \left[\begin{array}{cc}{b}_{1n}^{l}& {b}_{1n}^{u}\end{array}\right]\\ \cdots & \left[\begin{array}{cc}{b}_{2n}^{l}& {b}_{2n}^{u}\end{array}\right]\end{array}\\ \begin{array}{cc}\vdots & \vdots \\ \left[\begin{array}{cc}{b}_{m1}^{l}& {b}_{m1}^{u}\end{array}\right]& \left[\begin{array}{cc}{b}_{m2}^{l}& {b}_{m2}^{u}\end{array}\right]\end{array}& \begin{array}{cc}& \vdots \\ \cdots & \left[\begin{array}{cc}{b}_{mn}^{l}& {b}_{mn}^{u}\end{array}\right]\end{array}\end{array}\right]\end{array}$$

#### Step 2: Normalize matrices

*A* and *B* represent the matrix of design values and preference values, respectively. To compare and integrate *A* and *B* in the same way, we normalized both matrices with vector normalization. Equations of vector normalization are shown as follows, where [${x}_{ij}^{-},{x}_{ij}^{+}$] represents the normalized interval of [${x}_{ij}^{l},{x}_{ij}^{u}$].

38 $$\begin{array}{c}{x}_{ij}^{-}=\frac{{x}_{ij}^{l}}{\sqrt{{\sum }_{i=1}^{m}{\left({x}_{ij}^{u}\right)}^{2}}}\end{array}$$

39 $$\begin{array}{c}{x}_{ij}^{+}=\frac{{x}_{ij}^{u}}{\sqrt{{\sum }_{i=1}^{m}{\left({x}_{ij}^{u}\right)}^{2}}}\end{array}$$

Used with Eqs. (38) and (39), the normalized design value matrix ${A}^{\mathrm{^{\prime}}}={\left(\left[{a}_{ij}^{-},{a}_{ij}^{+}\right]\right)}_{m\times n}$ and the normalized preference value matrix ${B}^{\mathrm{^{\prime}}}={\left([{b}_{ij}^{-},{b}_{ij}^{+}]\right)}_{m\times n}$ are established.

#### Step 3: Integrate information

In this step, the normalized matrices are integrated. First, we introduce a coefficient $\mu \in [\mathrm{0,1}]$ to illustrate the contribution of design values and preference values.

Then we have

40 $$\begin{array}{c}\left[{c}_{ij}^{-},{c}_{ij}^{+}\right]=\left\{\begin{array}{c}\left(1-\mu \right)\times \left[{a}_{ij}^{-},{a}_{ij}^{+}\right]+\mu \times \left[{b}_{ij}^{-},{b}_{ij}^{+}\right] Both\, design\, value\, and\, expert\, preference\, available\\ \left[{b}_{ij}^{-},{b}_{ij}^{+}\right] Only\, expert\, preference\, available \\ \left[{a}_{ij}^{-},{a}_{ij}^{+}\right] Only\, design\, value\, available \end{array}\right.\end{array}$$

It is obvious that when $\mu >0.5$, the expert preference is considered to be superior in the evaluation. In contrast, $\mu >0.5$ reveals the design value is more significant. $\mu =0.5$ means information from both sources has equal importance.

Finally, the decision matrix is formed as

41 $$\begin{array}{c}C={\left(\left[{c}_{ij}^{-},{c}_{ij}^{+}\right]\right)}_{m\times n}\end{array}$$

### Phase 3: Determine interval entropy weight

Shannon^63^ proposed the entropy theory to quantify the information. In the decision-making process, information is used to rank the alternatives. Lotfi^42^ introduced an interval Shannon entropy approach, and implemented the interval entropy in MADM. Chen^4^ applied the interval entropy method in product concept evaluation. The interval weight can be calculated by the steps below.

Normalize the interval relative criterion importance rating [${p}_{j}^{-},{p}_{j}^{+}$] using the equations below:

42 $$\begin{array}{c}{p}_{ij}^{-}=\frac{{c}_{ij}^{-}}{{\sum }_{i=1}^{m}{c}_{ij}^{+}}\end{array}$$

43 $$\begin{array}{c}{p}_{ij}^{-}=\frac{{c}_{ij}^{+}}{{\sum }_{i=1}^{m}{c}_{ij}^{+}}\end{array}$$

We set $\left[{H}_{j}^{-} {H}_{j}^{+}\right]$ to satisfy the properties for ${p}_{j}$. The entropy constant equals $1/(\mathrm{ln}m)$, ${H}_{j}^{-}$ and ${H}_{j}^{+}$ can be expressed as:

44 $$\begin{array}{c}{H}_{j}^{-}=\mathrm{min}\left\{\frac{-\left[{\sum }_{i=1}^{m}{p}_{ij}^{-}\mathrm{ln}\left({p}_{ij}^{-}\right)\right]}{\mathrm{ln}\left(m\right)},\frac{-\left[{\sum }_{i=1}^{m}{p}_{ij}^{+}\mathrm{ln}\left({p}_{ij}^{+}\right)\right]}{\mathrm{ln}\left(m\right)}\right\}\end{array}$$

45 $$\begin{array}{c}{H}_{j}^{+}=\mathrm{max}\left\{\frac{-\left[{\sum }_{j=1}^{m}{p}_{ij}^{-}\mathrm{ln}\left({p}_{ij}^{-}\right)\right]}{\mathrm{ln}\left(m\right)},\frac{-\left[{\sum }_{j=1}^{m}{p}_{ij}^{+}\mathrm{ln}\left({p}_{ij}^{+}\right)\right]}{\mathrm{ln}\left(m\right)}\right\}\end{array}$$

In the equations above, when ${p}_{ij}^{-}=0$, we set ${p}_{ij}^{-}\mathrm{ln}\left({p}_{ij}^{-}\right)=0$. Similarly, when ${p}_{ij}^{+}=0$, $p_{ij}^{ + } {\text{ln}}\left( {p_{ij}^{ + } } \right) = 0$.

The lower and the upper bound using the interval weight of attribute $j$ can be computed by the following equations.

Lower bound:

46 $$\begin{array}{c}{w}_{j}^{-}=\frac{1-{H}_{j}^{+}}{{\Sigma }_{i=1}^{n}\left(1-{H}_{j}^{-}\right)}\end{array}$$

Upper bound:

47 $$\begin{array}{c}{w}_{j}^{+}=\frac{1-{H}_{j}^{-}}{{\Sigma }_{i=1}^{n}\left(1-{H}_{j}^{+}\right)}\end{array}$$

The criterion weight interval can be expressed as ${[w}_{j}^{-}, {w}_{j}^{+}]$.

### Phase 4: Rank the alternatives by the rough-TOPSIS method

In the previous sections, the information matrix $C$ with RNs and the interval criterion weight ${[w}_{j}^{-}, {w}_{j}^{+}]$ were prepared. In this section, the alternatives are ranked based on the rough-TOPSIS method. The steps are as follows.

*Step 1*: Determine the weighted normalized rough matrix $V={\left(\left[{v}_{ij}^{-},{v}_{ij}^{+}\right]\right)}_{m\times n}$ with the equation below.

48 $$\begin{array}{c}\left[{v}_{ij}^{-},{v}_{ij}^{+}\right]=\left[{w}_{j}^{-}, {w}_{j}^{+}\right]\times \left[{c}_{ij}^{-},{c}_{ij}^{+}\right]\end{array}$$

*Step 2*: Calculate the PIS ${v}_{P}\left(j\right)$ and the NIS ${v}_{N}\left(j\right)$ with the following equations:

49 $$\begin{array}{c}{v}_{P}\left(j\right)=\left\{\underset{i}{\mathrm{min}}\left({v}_{ij}^{-}\right),j\in \mathrm{Benefit\, attribute}; \underset{i}{\mathrm{max}}\left({v}_{ij}^{+}\right), j\in \mathrm{Cost\, attribute}\right\}\end{array}$$

50 $$\begin{array}{c}{v}_{N}\left(j\right)=\left\{\underset{i}{\mathrm{max}}\left({v}_{ij}^{+}\right),j\in \mathrm{Benefit\, attribute}; \underset{i}{\mathrm{min}}\left({v}_{ij}^{-}\right), j\in \mathrm{Cost\, attribute}\right\}\end{array}$$

*Step 3*: Compute the distance between the PIS and $\left[{v}_{ij}^{-},{v}_{ij}^{+}\right]$ in the normalized matrix $[{d}_{Pij}^{-},{d}_{Pij}^{+}]$ by the equations below:

51 $$\begin{array}{c}{d}_{Pij}^{-}=\left\{\begin{array}{cc}{v}_{P}\left(j\right)-{v}_{ij}^{+}& benefit\, attribute\\ {v}_{ij}^{-}-{v}_{P}\left(j\right)& cost\, attribute \end{array}\right.\end{array}$$

52 $$\begin{array}{c}{d}_{Pij}^{+}=\left\{\begin{array}{cc}{v}_{P}\left(j\right)-{v}_{ij}^{-}& benefit\, attribute\\ {v}_{ij}^{+}-{v}_{P}\left(j\right)& cost\, attribute \end{array}\right.\end{array}$$

Similarly, the distance between NIS and $\left[{v}_{ij}^{-},{v}_{ij}^{+}\right]$ in the normalized matrix $[{d}_{Nij}^{-},{d}_{Nij}^{+}]$ can be computed by the equations below:

53 $$\begin{array}{c}{d}_{Nij}^{-}=\left\{\begin{array}{cc}{v}_{ij}^{-}-{v}_{N}\left(j\right)& benefit\, attribute\\ {v}_{N}\left(j\right)-{v}_{ij}^{+}& cost\, attribute \end{array}\right.\end{array}$$

54 $$\begin{array}{c}{d}_{Nij}^{+}=\left\{\begin{array}{cc}{v}_{ij}^{+}-{v}_{N}\left(j\right)& benefit\, attribute\\ {v}_{N}\left(j\right)-{v}_{ij}^{-}& cost\, attribute \end{array}\right.\end{array}$$

*Step 4*: Determine the total distance of alternative $i$ to PIS ${D}_{Pi}=[{D}_{Pi}^{-},{D}_{Pi}^{+}]$ and NIS ${D}_{Ni}=[{D}_{Ni}^{-},{D}_{Ni}^{+}]$ by the following equations:

55 $$\begin{array}{c}{D}_{Pi}^{-}=\sqrt{\sum_{j=1}^{n}{\left({d}_{Pij}^{-}\right)}^{2}}\end{array}$$

56 $$\begin{array}{c}{D}_{Pi}^{+}=\sqrt{\sum_{j=1}^{n}{\left({d}_{Pij}^{+}\right)}^{2}}\end{array}$$

57 $$\begin{array}{c}{D}_{Ni}^{-}=\sqrt{\sum_{j=1}^{n}{\left({d}_{Nij}^{-}\right)}^{2}}\end{array}$$

58 $$\begin{array}{c}{D}_{Ni}^{+}=\sqrt{\sum_{j=1}^{n}{\left({d}_{Nij}^{+}\right)}^{2}}\end{array}$$

*Step 5*: Use the optimistic indicator $\alpha \in [\mathrm{0,1}]$ here^13^. A high $\alpha$ value ($\alpha >0.5$) indicates that the decision makers are more optimistic; vice versa, a low value ($\alpha <0.5$) expresses the decision makers’ pessimism. Normally, the value $\alpha$ is 0.5 for rational decision makers. The computing equations are:

59 $$\begin{array}{c}{D}_{Pi}^{*}=\left(1-\mathrm{\alpha }\right){D}_{Pi}^{+}+\alpha {D}_{Pi}^{-}\end{array}$$

60 $$\begin{array}{c}{D}_{Ni}^{*}=\left(1-\mathrm{\alpha }\right){D}_{Ni}^{+}+\alpha {D}_{Ni}^{-}\end{array}$$

The distance closeness indices of alternative $i$ (${CI}_{i}$) can be determined by the following equation:

61 $$\begin{array}{c}{CI}_{i}=\frac{{D}_{Ni}^{*}}{{D}_{Pi}^{*}+{D}_{Ni}^{*}}\end{array}$$

The alternative with a larger ${D}_{Ni}^{*}$ and a smaller ${D}_{Pi}^{*}$ is a better choice in decision-making. Hence, the alternative $i$ whose ${CI}_{i}$ approaches 1 is an optimal candidate, and the alternatives can be ranked by the value of $CIs$.

### Informed consent

No informed consent was required, because the data are anonymized.

## Case study

In our study, product concept evaluation of a cruise ship passenger cabin is used to illustrate the application of our method in a real-life case study. Three cabin design schemes $\{{A}_{1},{A}_{2},{A}_{3}\}$ have been generated by designers as the alternatives. The evaluation objective is to select the optimal scheme out of the three alternatives.

Previous customer information^45^ reveals the passenger cabin should be comfortable, aesthetic and eco-friendly. Therefore, in order to meet the requirements of the passengers while considering all the aspects of the design, nine criteria are identified by the decision-making organizers.

The design criteria ${C}_{1}$ to ${C}_{9}$ are as follows. ${C}_{1}$: Size, ${C}_{2}$: User acceptance, ${C}_{3}$: Ergonomics and design humanized, ${C}_{4}$: Style and trend, ${C}_{5}$: Reasonable placement of furniture, ${C}_{6}$: Innovation and competitiveness, ${C}_{7}$: Luxurious feeling, ${C}_{8}$: Eco-friendly, and ${C}_{9}$: Cost and economical. Among the nine criteria, ${C}_{1}$ and ${C}_{9}$ are determined by both design values and preference values, ${C}_{2}$ to ${C}_{8}$ are not available to get as design values and ${C}_{9}$ is the only cost attribute.

### Product concept evaluation by proposed method

#### Phase 1: Determine cluster weights

Thirty experts are selected as the decision makers with 10 members in each of the three clusters: the designer cluster (DC, marked as cluster 1), the manufacturer cluster (MC, marked as cluster 2) and the customer cluster (CC, marked as cluster 3). The decision makers are assigned to give their preferences according to each attribute.

Before the preference data is treated, the experts are required to make a pairwise comparison among the clusters. Then we calculated the cluster judgment by the arithmetic mean. The cluster pairwise comparisons are shown in Table 4, where cells shaded in grey mean being unable to compare against itself, and the symbol “N/A” means the corresponding cell is not permitted to compare with others.

**Table 4:**
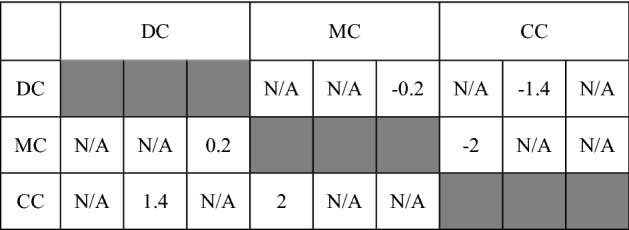
Pairwise comparisons of clusters.

*DC: designer cluster; MC: manufacturer cluster; CC: customer cluster.

Equations (26)–(28) can be simplified as:

62 $$\begin{array}{c}3{\mathrm{w}}_{\alpha }-{\sum }_{\beta =1,\beta \ne \alpha }^{3}{\mathrm{w}}_{\beta }={\sum }_{\beta =1,\beta \ne \alpha }^{3}{\sum }_{y=1,y\ne \alpha }^{3}{q}_{\alpha \beta y},\alpha =\mathrm{1,2},3\end{array}$$

In Eq. (29), ${\mathrm{q}}_{\alpha \beta y}=\upgamma {\updelta }_{\alpha \beta y}$, we have the equation set

63 $$\begin{array}{c}\left\{\begin{array}{cc}2{\mathrm{w}}_{1}-{\mathrm{w}}_{2}-{\mathrm{w}}_{3}=\upgamma {\sum }_{\beta =1,\beta \ne 1}^{3}{\sum }_{y=1,y\ne 1}^{3}{\updelta }_{1\beta y}=\left(1.4+0.2\right)\upgamma & \alpha =1\\ -{\mathrm{w}}_{1}+2{\mathrm{w}}_{2}-{\mathrm{w}}_{3}=\upgamma {\sum }_{\beta =1,\beta \ne 2}^{3}{\sum }_{y=1,y\ne 2}^{3}{\updelta }_{2\beta y}=\left(2-0.2\right)\upgamma & \alpha =2\\ -{\mathrm{w}}_{1}-{\mathrm{w}}_{2}+2{\mathrm{w}}_{3}=\upgamma {\sum }_{\beta =1,\beta \ne 3}^{3}{\sum }_{y=1,y\ne 3}^{3}{\updelta }_{3\beta y}=\left(-2-1.4\right)\upgamma & \alpha =3\end{array}\right.\end{array}$$

${\mathrm{w}}_{1}$ to ${\mathrm{w}}_{3}$ are calculated as:

$${\mathrm{w}}_{1}=\frac{8}{15}\upgamma ; {\mathrm{w}}_{2}=\frac{9}{15}\upgamma ; {\mathrm{w}}_{3}=-\frac{17}{15}\upgamma.$$

The cluster weights are:

$${W}_{DC}=0.423; {W}_{MC}=0.443; {W}_{CC}=0.423.$$

#### Phase 2: Normalize and integrate decision matrices

Next, after the linguistic preferences are transformed into crisp numbers on the 7-level scale listed in Sect. 3.2, the crisp numbers are converted into rough numbers by Eqs. (1)–(5). Taking the first expert in the designer cluster as an example, the corresponding data are shown in Table 5.

**Table 5:** Preference values and corresponding limits of interval ${{\varvec{U}}}_{{\varvec{t}}}^{{\varvec{l}}}$ and ${{\varvec{U}}}_{{\varvec{t}}}^{{\varvec{u}}}$ of the designer cluster DC.

Expert t in DC	${C}_{1}$ of A_1_	${U}_{t}^{l}$	${U}_{t}^{u}$
Designer 1	7	6.000	7.000
Designer 2	6	5.571	6.375
Designer 3	6	5.571	6.375
Designer 4	6	5.571	6.375
Designer 5	6	5.571	6.375
Designer 6	4	4.000	6.000
Designer 7	7	6.000	7.000
Designer 8	6	5.571	6.375
Designer 9	7	6.000	7.000
Designer 10	5	4.500	6.222
Average	N/A	5.436	6.510

Similarly, the preferences of the three clusters (DC, MC and CC) are converted into rough numbers, and the integrated interval of alternative ${A}_{1}$ is determined, as shown in Table 6.

**Table 6:** Step data based on Eqs. (33)–(35).

	${U}_{\alpha }^{l}$	${U}_{\alpha }^{u}$	${W}_{\alpha }$
DC	5.436	6.51	0.423
MC	5.493	6.313	0.443
CC	4.937	5.652	0.133
Integrated	5.389	6.302	

The matrix of preference values and normalized data based on Eqs. (38) and (39) are shown in Table 7.

**Table 7:** Step data of preference value (PV) matrix.

Attribute	A1	A2	A3	Normalized A1	Normalized A2	Normalized A3
${C}_{1}$	[5.389,6.302 ]	[4.767,5.741 ]	[5.035,5.979 ]	[0.518,0.605 ]	[0.458,0.551 ]	[0.484,0.574 ]
${C}_{2}$	[4.324,5.552 ]	[4.524,5.841 ]	[4.507,5.781 ]	[0.436,0.560 ]	[0.456,0.589 ]	[0.454,0.583 ]
${C}_{3}$	[5.409,6.205 ]	[4.376,5.873 ]	[4.992,6.070 ]	[0.516,0.592 ]	[0.418,0.560 ]	[0.476,0.579 ]
${C}_{4}$	[4.618,5.280 ]	[4.677,5.515 ]	[4.507,5.174 ]	[0.501,0.572 ]	[0.507,0.598 ]	[0.489,0.561 ]
${C}_{5}$	[4.237,5.685 ]	[5.573,6.463 ]	[5.554,6.550 ]	[0.392,0.526 ]	[0.515,0.598 ]	[0.514,0.606 ]
${C}_{6}$	[4.011,5.508 ]	[4.558,5.783 ]	[4.569,5.745 ]	[0.408,0.560 ]	[0.463,0.588 ]	[0.464,0.584 ]
${C}_{7}$	[4.575,5.566 ]	[5.197,5.939 ]	[4.944,5.729 ]	[0.460,0.559 ]	[0.522,0.597 ]	[0.497,0.576 ]
${C}_{8}$	[5.074,5.916 ]	[5.529,6.339 ]	[5.150,6.250 ]	[0.475,0.554 ]	[0.517,0.593 ]	[0.482,0.585 ]
${C}_{9}$	[4.218,5.145 ]	[4.662,5.561 ]	[4.864,6.084 ]	[0.434,0.529 ]	[0.480,0.572 ]	[0.501,0.626 ]

In this case, the attributes ${C}_{1}$ and ${C}_{9}$ are available to obtain the corresponding design values. The design values are normalized by Eqs. (1) to (5), and the design value matrix is shown as Table 8.

**Table 8:** Normalized design values (DV) matrix.

Attribute	A1	A2	A3
${C}_{1}$	[0.553,0.571]	[0.571,0.580]	[0.571,0.580]
${C}_{2}$	N/A	N/A	N/A
${C}_{3}$	N/A	N/A	N/A
${C}_{4}$	N/A	N/A	N/A
${C}_{5}$	N/A	N/A	N/A
${C}_{6}$	N/A	N/A	N/A
${C}_{7}$	N/A	N/A	N/A
${C}_{8}$	N/A	N/A	N/A
${C}_{9}$	[0.564,0.590]	[0.551,0.577]	[0.538,0.564]

Using Eqs. (40) and (41), let $\mu =0.5$, the decision matrix can be determined.

#### Phase 3: Determine interval entropy weight

Subsequently, the entropy weights are determined by Eqs. (42) to (47), the weight of criteria calculated. The decision matrix and criteria are shown in Table 9.

**Table 9:** The interval weight of criteria.

	Bound	${C}_{1}$	${C}_{2}$	${C}_{3}$	${C}_{4}$	${C}_{5}$	${C}_{6}$	${C}_{7}$	${C}_{8}$	${C}_{9}$
A1	Lower limit	0.535	0.436	0.516	0.501	0.392	0.408	0.460	0.475	0.499
	Upper limit	0.588	0.560	0.592	0.572	0.526	0.560	0.559	0.554	0.560
A2	Lower limit	0.514	0.456	0.418	0.507	0.515	0.463	0.522	0.517	0.516
	Upper limit	0.566	0.589	0.560	0.598	0.598	0.588	0.597	0.593	0.575
A3	Lower limit	0.527	0.454	0.476	0.489	0.514	0.464	0.497	0.482	0.519
	Upper limit	0.577	0.583	0.579	0.561	0.606	0.584	0.576	0.585	0.595
Criteria weight	Lower limit	0.920	0.943	0.902	1.057	1.070	0.949	0.843	1.061	1.279
	Upper Limit	0.999	1.064	1.027	1.197	1.178	1.143	0.935	1.288	1.641

#### Phase 4: Rank the alternatives by the rough-TOPSIS method

Finally, the relative variables and *CI*s are computed by Eqs. (36) to (42), indicated in Table 10. The best design concept based on design values and expert preferences is A2, and the ranking of the alternatives can be calculated by the *CI*s, which is $A2\succ A3\succ A1$.

**Table 10:** The relative variables and *CI*s.

	${D}_{Pi}^{*}$	${D}_{Ni}^{*}$	$CI$
A1	0.231	0.216	0.484
A2	0.189	0.250	0.570
A3	0.199	0.244	0.550

### Further analysis

To show the influence of the modifications, we made comparisons on the proposed method, the rough-TOPSIS method without the expert weight consideration and the rough-TOPSIS method without the information integration. In our study, the rough-TOPSIS method with rough-entropy criteria weight is applied in the assessment process. This process has been proven effective in design concept evaluation^4,12,13,16,53^. Hence, in this section, we focus on the sensitivity analysis of the proposed method.

**Figure 5:**
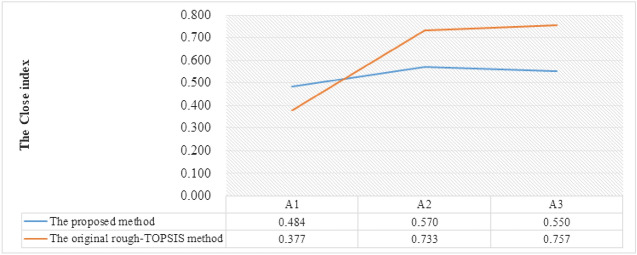
The comparison between the original rough-TOPSIS method and the proposed method.

Firstly, a comparison is proposed to reveal the effectiveness of the proposed method. In our study, a rough-entropy criteria weight-based assessment is implemented, and the differences between the original rough-TOPSIS method and the proposed method are the criteria weights, which are shown in Table 11. The CIs of the original rough-TOPSIS method with entropy criteria weight and the proposed method are calculated, as shown in Fig. 5. It is obvious the CI of $A1$ is inferior compared to the other two design concepts, no matter whether using the proposed method or the original rough-TOPSIS method. However, the optimal alternative in the rankings varies because of the different criteria weights. In the original rough-TOPSIS method, the ranking is $A3\succ A2\succ A1$ while the ranking in the proposed method is $A2\succ A3\succ A1$. In the design concept evaluation, the rough number information shows the uncertainty of the experts’ preference, which means the data may fluctuate in an interval. Thus, the entropy weight which is generated from the decision maker’s preference is preferred to be an interval as well. Hence, the rough-entropy criteria weight-based assessment is more practical for design concept evaluation.

**Table 11:** The criteria weight of the rough-TOPSIS method and the proposed method.

Criteria weight	C1	C2	C3	C4	C5	C6	C7	C8	C9
The ${w}_{j}^{-}$ of the proposed method	0.920	0.943	0.902	1.057	1.070	0.949	0.843	1.061	1.279
The ${w}_{j}^{+}$ of the proposed method	0.999	1.064	1.027	1.197	1.178	1.143	0.935	1.288	1.641
Criteria weight of rough-TOPSIS method	0.010	0.035	0.162	0.041	0.498	0.094	0.088	0.061	0.011

**Figure 6:**
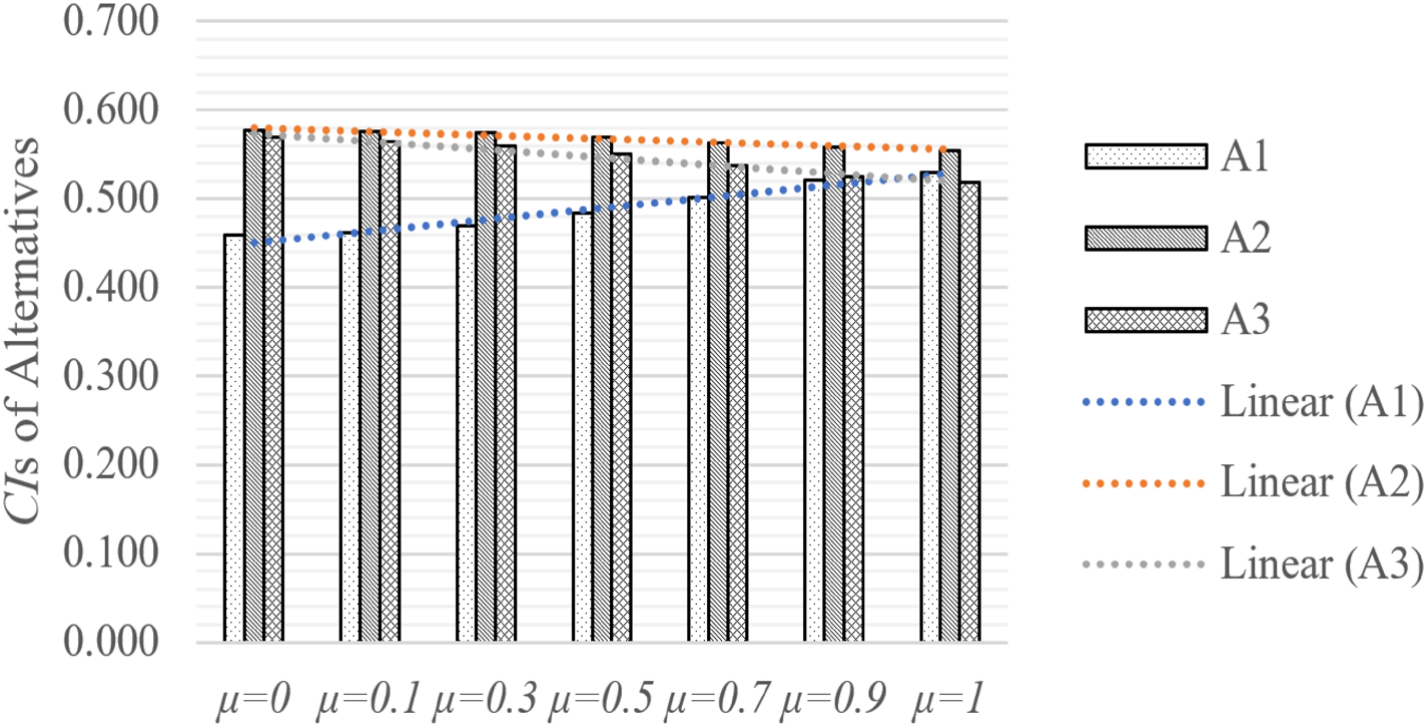
Closeness indices (CIs) of alternatives by different coefficient *μ.*

In Phase 3, the integrated matrix is determined by the coefficient $\mu$. The coefficient shows the contribution of design values and preference values in the decision matrix, and the relative coefficient $\mu$ and the corresponding CI are shown in Fig. 6. When $\mu =0$, the element decision matrix is only determined by design values. Similarly, when $\mu =1$, the element decision matrix is only determined by preference values. We can infer from the figure that the ranking of alternatives does not change as while the contribution coefficient $\mu$ changes from 0 to 0.9, the preference remains $A2\succ A3\succ A1$. It is obvious that while $\mu$ increases from 0 to 1, $A1$ and $A2$ decline while $A3$ increases. What we need to notice is that, when $\mu$ equals 0.9, the CI of $A1$ and $A3$ are very close, with a value of 0.521 and 0.524, respectively. When the coefficient reaches 1, the result is calculated by all the preference values, and the ranking of the alternatives changes to $A2\succ A1\succ A3$.

**Figure 7:**
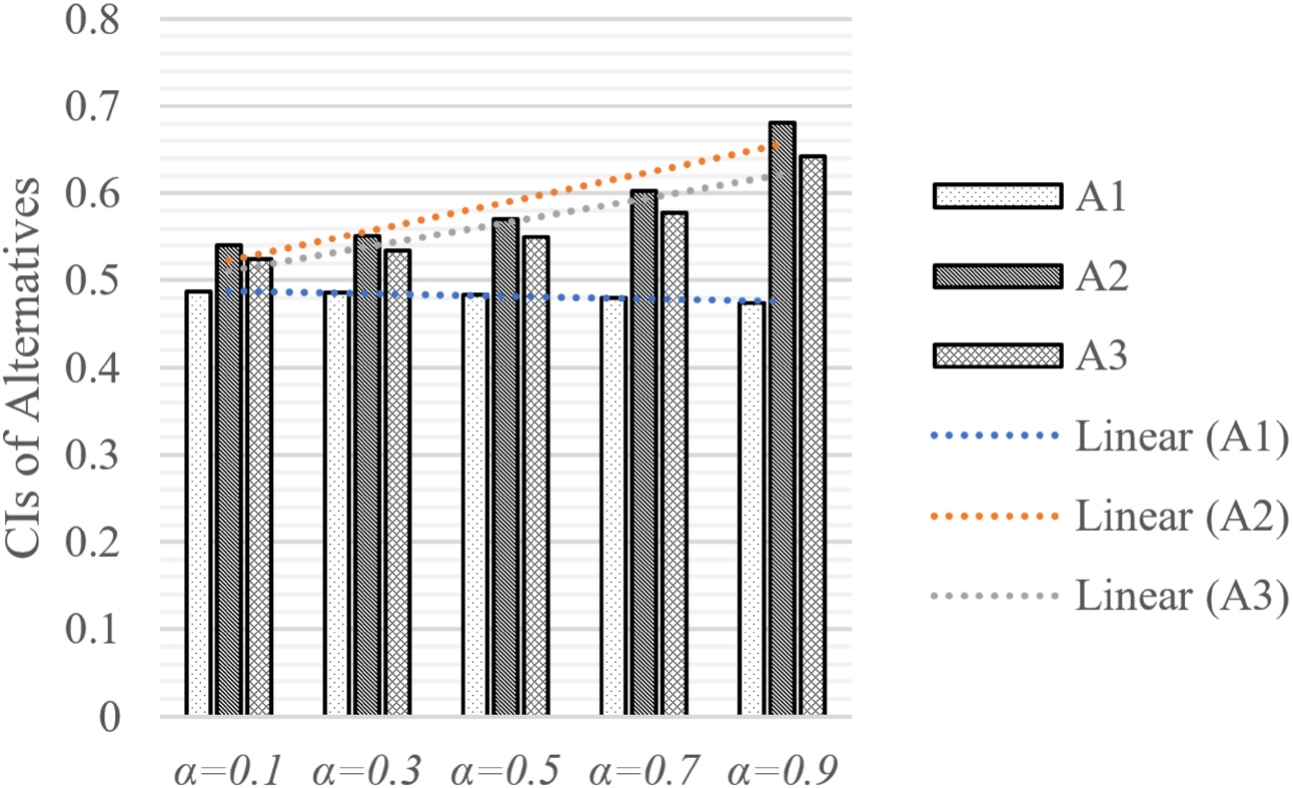
Closeness indices (CIs) of alternatives by different coefficient *α.*

We also compared the different optimism levels (*α*) of decision makers. The alternative ranking is calculated as shown in Fig. 7. While *α* increases from 0.1 to 0.9, the design alternative ranking remains in the same sequence, $A2\succ A3\succ A1$. From the variance tendency, we can see that as the optimism level increases, both $A2$ and $A3$ increase. On the contrary, the most negative alternative $A1$ declines, and the gap between $A1$ and the other two alternatives increases in this process.

As a comparison, we applied the same rough-TOPSIS method without cluster weight determination. The experts are regarded as homogeneous individuals, and the ranking of the alternatives is shown in Table 12. Without cluster weight, the sequence of the alternatives may vary. In our case, when the contribution α chooses 0.1 or 0.3, alternative 3 is the optimal option, which is different from the result considering cluster weight.

**Table 12:** Alternative rankings with and without cluster weight considered.

	α = 0.1	α = 0.3	α = 0.5	α = 0.7	α = 0.9
Ranking without cluster weight	$A3\succ A2\succ A1$	$A3\succ A2\succ A1$	$A2\succ A3\succ A1$	$A2\succ A3\succ A1$	$A2\succ A3\succ A1$
Ranking with cluster weight	$A2\succ A3\succ A1$	$A2\succ A3\succ A1$	$A2\succ A3\succ A1$	$A2\succ A3\succ A1$	$A2\succ A3\succ A1$

Hence, we can infer from the comparative analysis that integrating design values and preference values makes the evaluation more accurate. Ignoring design values or expert preferences may lead to a different ranking. Moreover, considering the cluster weight can also help the project manager to eliminate or reduce the influence of the different backgrounds of various experts.

## Conclusion

As an effective approach in design concept evaluation the rough-TOPSIS method reveals excellent performance in the ambiguity and imprecision of the evaluation of complicated product design concepts. This paper provides a modified rough-TOPSIS method. Two modifications are presented in this study:

1. Consideration of expert weight. We classified the experts into three clusters: the designer cluster, the manufacturer cluster and the customer cluster. The expert weights are considered by a cluster weight determination method. The cluster weights are determined by a Multiplicative AHP method.
2. Preservation of information from the design values and the expert preferences. We introduced a 3-step process with a coefficient $\mu$ to represent the contribution of the two sources. Both information sources are integrated and formed a hybrid decision matrix.

Application and comparison based on the proposed method were implemented. The result shows it is a feasible method for design concept evaluation. Further analysis indicates both cluster weight and the source of the information may affect the result of the decision making, and our modifications in design concept evaluation may improve the precision of the result.

Although the proposed method is shown to be an effective MADM model in design concept evaluation, some improvements can be made in future study. The coefficient $\mu$ for information integration may not represent different attributes, and a dynamic variable can better illustrate real situations. Applications in other fields also need to be verified by real-world applications.

### Ethics approval

This article does not contain any studies with human participants or animals performed by any of the authors.

## Supplementary Information

Supplementary Information.

## Data Availability

The data that supports the findings of this study are available in the supplementary material of this article.
