# Supplementary material for: A novel integrated MADM method for design concept evaluation
Source: Sci Rep. 2022 Sep 23;12:15885. doi: 10.1038/s41598-022-20044-7 (PMC9508270; doi:10.1038/s41598-022-20044-7)
Supplement: Supplementary file 1 — Supplementary Information. [file 41598_2022_20044_MOESM1_ESM.pdf]

Appendix 1: Pairwise comparison of clusters

Designer	D/M	D/C	M/C	Manufacturer	D/M	D/C	M/C	Customer	D/M	D/C	M/C
D1	4	2	2	M1	0	2	2	C1	-2	2	4
D2	2	4	2	M2	0	2	0	C2	0	-2	-2
D3	2	6	2	M3	-2	0	0	C3	-2	0	-2
D4	0	2	0	M4	0	2	4	C4	2	2	0
D5	2	4	2	M5	-2	0	0	C5	0	-2	-2
D6	2	4	2	M6	-2	0	2	C6	4	2	2
D7	0	2	2	M7	0	2	2	C7	2	0	0
D8	0	4	4	M8	2	4	2	C8	0	-2	0
D9	2	4	2	M9	0	0	0	C9	0	2	0
D10	0	4	2	M10	-2	2	0	C10	-2	0	0

*D: designer cluster; M: manufacturer cluster; C: customer cluster

Appendix 2: Design values

\Criteria Alternative	C1	C2	C3	C4	C5	C6	C7	C8	C9
	Size	User acceptance	Ergonomics	design humanized	Reasonable placement of furniture	Innovation and competitiveness	Luxurious feeling	Eco-friendly	Cost and economical
Type	Benefit	Benefit	Benefit	Benefit	Benefit	Benefit	Benefit	Benefit	Cost
A1	20	N/A	N/A	N/A	N/A	N/A	N/A	N/A	53.5
A2	21	N/A	N/A	N/A	N/A	N/A	N/A	N/A	51.2
A3	21	N/A	N/A	N/A	N/A	N/A	N/A	N/A	48.8

Appendix 3: Pereference values of alternative 1

Criteria		Size	User acceptance	Ergonomics	design humanized	Reasonable placement of furniture	Innovation and competitivene	Luxurious feeling	Eco-friendly	Cost and economical
Type		Benefit	Benefit	Benefit	Benefit	Benefit	Benefit	Benefit	Benefit	Cost
Designer Cluster	D1	7	6	6	7	5	4	4	6	4
	D2	6	4	7	7	6	5	6	7	4
	D3	6	4	6	7	3	3	6	5	6
	D4	6	7	5	6	5	7	6	4	3
	D5	6	5	6	4	6	4	7	6	5
	D6	4	4	5	4	4	7	4	4	3
	D7	7	5	5	7	6	4	6	6	5
	D8	6	7	7	5	7	5	6	5	4
	D9	7	6	6	7	5	6	5	6	5
	D10	5	3	4	4	3	5	6	5	5
Manufacturer Cluster	M1	6	5	5	4	6	5	4	5	4
	M2	5	4	5	5	5	4	4	6	5
	M3	7	4	6	5	6	4	5	5	5
	M4	5	4	6	6	6	3	4	6	6
	M5	6	5	6	5	5	6	3	5	5
	M6	5	5	6	6	4	4	4	5	5
	M7	6	5	7	5	5	6	5	6	5
	M8	7	6	6	5	4	5	6	6	4
	M9	6	5	6	6	4	4	5	6	5
	M10	6	5	6	6	3	3	4	6	4
Customer Cluster	C1	5	4	6	4	5	5	4	6	5
	C2	6	5	5	5	5	4	6	6	6
	C3	6	6	6	7	5	6	5	5	7
	C4	4	6	6	6	6	6	5	4	5
	C5	5	4	7	4	6	4	6	5	5
	C6	5	6	6	6	7	6	6	7	4
	C7	6	5	6	6	4	5	6	5	5
	C8	6	4	6	6	5	4	7	5	6
	C9	5	3	5	5	5	4	6	6	4
	C10	5	6	6	5	7	7	6	6	5

Appendix 4: Pereference values of alternative 2

Criteria		Size	User acceptance	Ergonomics	design humanized	Reasonable placement of furniture	Innovation and competitivenes s	Luxurious feeling	Eco-friendly	Cost and economical
Type		Benefit	Benefit	Benefit	Benefit	Benefit	Benefit	Benefit	Benefit	Cost
Designer Cluster	D1	6	6	4	7	6	5	4	6	5
	D2	6	5	7	6	5	5	6	6	6
	D3	6	6	4	7	6	4	4	6	5
	D4	6	5	6	7	6	5	6	5	4
	D5	6	4	6	4	5	6	6	7	5
	D6	4	3	5	5	6	4	6	5	4
	D7	7	4	6	3	7	5	6	5	4
	D8	6	5	3	5	5	6	6	6	4
	D9	7	5	3	5	4	6	4	7	5
	D10	4	5	6	7	6	7	4	4	4
Manufacturer Cluster	M1	4	6	5	6	7	6	5	6	6
	M2	5	7	4	5	7	4	6	6	6
	M3	4	6	6	7	7	6	5	6	7
	M4	6	6	4	6	6	6	5	7	5
	M5	4	7	7	4	6	4	6	5	6
	M6	5	6	5	6	7	6	6	7	5
	M7	6	5	6	6	7	4	6	6	6
	M8	4	4	6	6	5	4	7	6	6
	M9	5	3	4	5	5	4	6	6	4
	M10	5	6	6	5	7	7	6	6	7
Customer Cluster	C1	6	6	5	6	7	6	5	6	6
	C2	6	7	4	5	6	4	6	7	4
	C3	4	6	6	7	6	6	6	6	4
	C4	6	5	4	5	6	4	6	7	5
	C5	4	5	7	4	6	4	6	5	6
	C6	5	6	5	6	7	6	6	7	4
	C7	6	5	5	5	6	4	6	6	4
	C8	4	4	4	6	5	5	7	6	4
	C9	5	3	4	5	6	4	6	6	4
	C10	5	6	6	5	7	7	6	6	4

Appendix 5 Pereference values of alternative 3

Criteria		Size	User acceptance	Ergonomics	design humanized	Reasonable placement of furniture	Innovation and competitivenes s	Luxurious feeling	Eco-friendly	Cost and economical
Type		Benefit	Benefit	Benefit	Benefit	Benefit	Benefit	Benefit	Benefit	Cost
Designer Cluster	D1	7	5	5	7	7	5	4	3	4
	D2	6	5	7	5	5	4	6	5	5
	D3	7	6	4	7	5	5	4	6	4
	D4	7	6	6	7	5	5	4	5	5
	D5	6	3	6	6	5	7	5	7	6
	D6	7	6	6	7	7	5	6	7	7
	D7	6	7	6	6	7	7	5	6	6
	D8	6	4	7	6	5	4	6	5	4
	D9	7	5	7	7	7	5	4	3	7
	D10	4	5	6	7	6	5	4	6	4
Manufacturer Cluster	M1	6	5	5	6	6	5	5	6	5
	M2	5	5	5	6	7	4	6	6	6
	M3	4	6	6	7	7	6	5	6	7
	M4	6	4	6	6	6	6	5	7	5
	M5	6	6	4	4	6	4	6	5	6
	M6	4	4	6	6	7	6	6	7	5
	M7	5	4	6	5	7	4	6	6	6
	M8	5	6	4	5	5	4	7	6	6
	M9	4	4	4	5	5	4	6	6	4
	M10	5	6	6	5	7	7	6	6	7
Customer Cluster	C1	4	6	5	6	7	6	5	6	6
	C2	5	7	4	5	4	4	5	5	5
	C3	4	6	6	7	6	6	5	6	5
	C4	6	6	4	6	6	6	5	7	6
	C5	4	7	7	4	6	4	6	5	5
	C6	5	6	5	6	7	6	6	7	6
	C7	6	5	6	6	6	4	6	6	6
	C8	4	4	6	6	5	6	5	5	6
	C9	5	3	4	5	5	6	6	5	6
	C10	5	6	6	5	6	7	6	6	5
